# PD-L1 deletion or blockade intrinsically reduces macrophage CD80 levels

**DOI:** 10.3389/fonc.2026.1917545

**Published:** 2026-10-30

**Authors:** Trinity Waddell, Haidong Dong, Minna Roh-Johnson, Jessica N. Lancaster

**Affiliations:** 1 Department of Immunology, Mayo Clinic, Phoenix, AZ, United States; 2 Department of Biochemistry, University of Utah, Salt Lake City, UT, United States; 3 Department of Immunology, Mayo Clinic, Rochester, MN, United States; 4 Department of Cancer Biology, Mayo Clinic, Phoenix, AZ, United States

**Keywords:** antigen presentation, checkpoint, macrophage, oncology, PD-L1, polarization, tumor microenvironment

## Abstract

**Introduction:**

Macrophages in the tumor microenvironment are known to upregulate PD-L1 expression, thereby suppressing T cells through PD-1 ligation. However, the manner in which PD-L1 expression intrinsically impacts macrophages and their immunomodulatory phenotype is less clear. Clarifying this knowledge gap may provide further insight into macrophage function within the tumor microenvironment.

**Methods:**

To characterize the macrophage intrinsic role of PD-L1, we used complementary genetic and pharmacological approaches by analyzing primary murine bone marrow-derived macrophages (BMDMs) with complete genetic PDL1 deletion and wildtype BMDMs treated with anti-PD-L1 blocking antibodies. Macrophages were evaluated in vitro across naïve, pro-inflammatory, and tumor conditioned polarization states.

**Results:**

Unlike prior reports, neither genetic deletion nor antibody blockade dramatically altered the expression of macrophage polarization markers or in vitro phagocytic capacity. However, both conditions consistently and robustly reduced surface levels of a costimulatory molecule, CD80. Reduced CD80 levels prompted further analysis of T cell interacting and antigen presentation associated surface proteins, in which we revealed disparate effects of expression in response to PD-L1 genetic deletion and antibody blockade.

**Discussion:**

Collectively, these findings suggest that loss of macrophage PD-L1 reduces CD80 expression and regulates immunoregulatory surface protein expression *in vitro*.

## Introduction

Strategies to successfully target tumor macrophages are needed to improve patient therapeutic responses. Macrophages are highly plastic innate immune cells that play an essential role in shaping the tumor microenvironment. Macrophages display a spectrum of polarization states, classically defined by a framework of pro- versus anti-inflammatory states, by which they facilitate myriad roles in infection, cancer, and tissue homeostasis (1–3). Cell-extrinsic and -intrinsic cues shape macrophage polarization states, which, in turn, enforce the context-specific activation states of T cells. Because of their immunomodulatory functions and their ability to take on unique polarization or functional profiles in response to local microenvironmental cues, macrophages play an important role in the efficacy of immunotherapy (4, 5).

The canonical function of programmed cell death-ligand 1 (PD-L1), a membrane-bound immune checkpoint ligand protein, is to bind the T cell inhibitory receptor programmed cell death protein 1 (PD-1), thereby suppressing T cell activation and adaptive immune responses (6–8). PD-L1 is frequently overexpressed on cancer cells as a mechanism of immune evasion, leading to suppression of T cell activation in the tumor microenvironment (9, 10). Immune checkpoint blockade therapies have been developed to interfere with the PD-1/PD-L1 axis with blocking antibodies to restore cytotoxic T cell function (11, 12). This therapeutic has had significant impact on patient outcomes in many types of cancers, underscoring the clinical relevance of PD-L1 (13).

While PD-L1 overexpression in cancer cells has been a primary target in PD-1/PD-L1 immune checkpoint blockade, PD-L1 expression by tumor-associated macrophages also plays a significant role in therapeutic outcomes (14–16). Prior work has indicated potential differential efficacies of PD-1 and PD-L1 blocking antibodies (17–20), suggesting that the cellular mechanisms driving outcomes of each monotherapy may differ. Despite its limited cytoplasmic domain, PD-L1 has been shown to have cell-intrinsic functions independent of PD-1 ligation, including intracellular and nuclear activities in a variety of cancer cell types (21, 22) and in dendritic cells (23). Connections between PD-L1 expression and macrophage activation status within tumors have also been noted (24). Subsequent studies in our lab (25) and others (17, 26–28) suggested that treatment of macrophages in monoculture with PD-L1 blocking antibodies promoted an immunostimulatory phenotype and function. These findings suggest that PD-L1 exerts function independent of the canonical PD-1/PD-L1 axis. However, the literature remains inconsistent as to whether PD-L1 expression intrinsically promotes or stabilizes macrophage states.

There currently remains a lack of understanding as to whether PD-L1 actively regulates macrophage activation or merely serves as a marker of macrophage activation state. PD-L1 expression is highest in M1 inflammatory macrophages and lowest in naïve macrophages (29). This is likely due to its upregulation induced by interferon-gamma (IFNγ) (30–32), but PD-L1 is also elevated in tumor conditioned (M2-like) macrophages over the naïve state. Because of the evidence that macrophage PD-L1 expression strongly influences the tumor immune environment, it is interesting to note the disparity in expression between inflammatory M1 and tumor conditioned macrophages. Though there has been some evidence of association, it is not yet clear whether PD-L1 influences macrophage polarization and ability to impact the immune microenvironment. This points to a critical need to better characterize PD-L1’s intrinsic roles in modulating macrophage functions independent of PD-1 interaction. Our goal was to address this challenge using genetic and pharmacological approaches. Towards this end, we compared primary murine macrophages with complete genetic abrogation of PD-L1 expression and those treated by antibody-mediated PD-L1 blockade against PD-L1-sufficient controls. For murine macrophages in isolation, we found that PD-L1 inhibition did not broadly impact macrophage polarization profiles or phagocytic capacities. However, PD-L1 inhibition robustly decreased CD80 protein levels without affecting transcript expression. Furthermore, our results indicated that abrogation and blockade of PD-L1 induced changes to macrophage expression of antigen presentation and immune checkpoint molecules in distinct ways. Taken together these data support a mechanism whereby PD-L1 targeting strategies exert their effects, at least in part, by modulating macrophage interactions within the tumor microenvironment.

## Results

### PD-L1 blockade and genetic abrogation have limited impact on macrophage polarization

We established an *in vitro* monoculture system in which we differentiated primary murine bone marrow derived macrophages (BMDMs) from C57BL/6J (PD-L1 WT) or B6.B7-H1^-/-^ (PD-L1 KO) mice (30). Naïve BMDMs (M0) were polarized toward a pro-inflammatory (M1) phenotype by the addition of IFNγ and lipopolysaccharide (LPS), or a tumor conditioned (TCM) state using interleukin-4 (IL-4), interleukin-10 (IL-10), and E0771 murine triple negative breast cancer cell line supernatant (**Figure 1A**). For the PD-L1 blockade group WT macrophages were treated with either anti-PD-L1 antibodies (clone 10F.9G2) or isotype controls during the 48-hour polarization step of our experimental workflow (**Figure 1A**). Macrophages were polarized for 48 hours consistent with prior literature (31, 32). PD-L1 deficiency and blockade were confirmed by detection of anti-PD-L1 surface staining with flow cytometry, and deficiency was further confirmed by both Western blot detection of whole PD-L1 protein from lysates of M1-polarized BMDMs and qPCR of RNA transcript levels (**Supplementary Figures 1A, B**).

**Figure 1:**
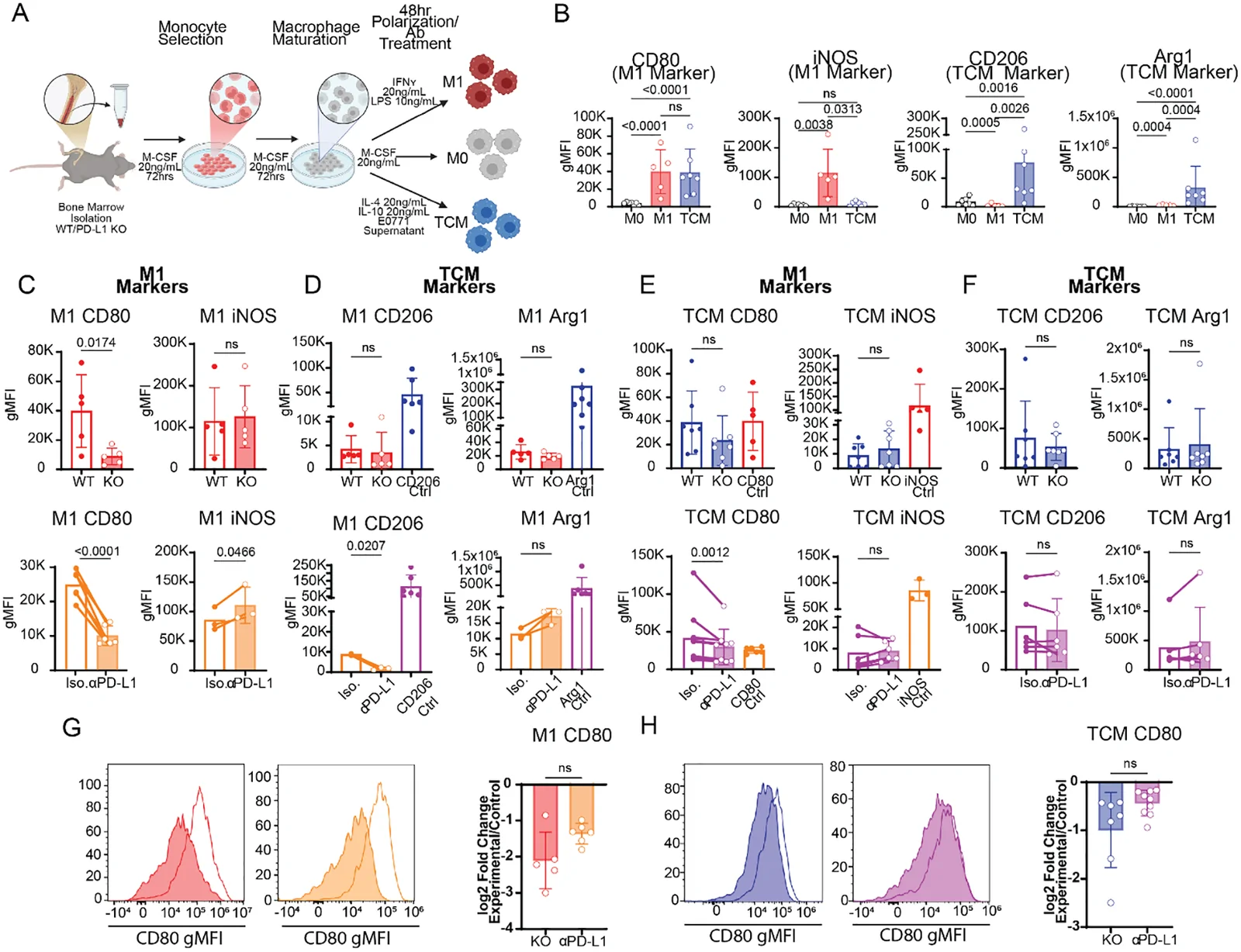
PD-L1 blockade and abrogation have limited impact on macrophage polarization. **(A)** Experimental setup: Bone marrow cells isolated from PD-L1-sufficient (WT) and -deficient (KO) mice were differentiated into naïve M0 BMDMs using M-CSF and polarized to M1 or TCM states by IFNγ/LPS or IL-4/IL-10/EO771 tumor medium for 48 h. WT Mice were also treated with either anti-PD-L1 antibody or isotype antibody during polarization. Legend for bar graph organization. Colors organized by Polarization: M1 Red and Orange, and TCM Blue and Purple, and by PD-L1 interference: WT/KO Red and Blue, Iso./Blockade Orange and Purple. **(B)** Flow cytometry Geometric mean fluorescence intensities (gMFI) of M1-associated (CD80, iNOS) and TCM-associated (CD206, Arg1) polarization markers in WT M0, M1, and TCM polarized BMDMs. M0 N = 5; M1 N = 6; TCM N = 7 mice. Data analysis performed with two-tailed multiple T-tests and mixed effects with Tukey’s multiple comparisons *post-hoc* analysis on log10 transformed data. **(C)** gMFI of M1 markers CD80 and iNOS upon M1 polarization of BMDMs comparing WT vs KO (N = 5 mice)(CD80 geometric mean fold change = 0.23 (95% CI 0.08 – 0.72)) and Isotype vs Blockade conditions. (CD80 N = 6 mice iNOS N = 3 mice)(CD80 and iNOS geometric mean fold change = 0.39 (95% CI 0.32 – 0.48), = 1.29 (95% CI 1.01 – 1.64)). **(D)** gMFI of TCM markers CD206 and Arg1 upon M1 polarization of BMDMs comparing WT vs KO (N = 5 mice) and Isotype vs Blockade (N = 3 mice) conditions. **(E)** gMFI of M1 markers CD80 and iNOS upon TCM polarization of BMDMs comparing WT vs KO (N = 7 mice) and Isotype vs Blockade (CD80 N = 9 mice, iNOS N = 6 mice) conditions (CD80 geometric mean fold change = 0.74 (95% CI 0.64 – 0.85)). **(F)** gMFI of TCM markers CD206 and Arg1 upon TCM polarization of BMDMs comparing WT vs KO (N = 7 mice) and Isotype vs Blockade conditions (N = 6 mice). **(G)** Fold change in CD80 expression of M1 polarized BMDMs comparing KO over WT and Blockade over Isotype treatment. (KO N = 5 mice, Blockade N = 6 mice). **(H)** Fold change in CD80 expression of TCM polarized BMDMs comparing KO over WT and Blockade over Isotype treatment. (KO N = 7 mice Blockade N = 9 mice). Each dot represents a biological replicate. Each biological replicate represents the mean of a technical triplicate derived from one mouse. All conditions were repeated on at least 2 experimental days with the exception of markers iNOS, CD206, and Arg1 in M1 polarized Isotype vs Blockade groups. Two-tailed Welch’s corrected T-tests were used on log10 transformed gMFI to compare WT vs KO. Control columns correspond to the associated polarization state and are shown for biological context, not included in analysis. Two-tailed Paired T-tests were used on log10 transformed gMFI to compare isotype vs anti-PD-L1 treated. Control columns correspond to the associated polarization state are shown for biological context, not included in analysis. Due to unmixing of fluorescent signals a constant value was used to ensure all samples expressed a positive fluorescent signal. The constant was applied to all samples in the condition and always resulted in the lowest expressing sample having a signal of 1000. A constant of 3282 and 1487.333 were applied to panel D WT/KO CD206 and Iso./aPD-L1 CD206 respectively. Two-tailed Welch’s corrected T-tests were used to compare KO and anti-PD-L1 treated fold changes in G and H.

The baseline phenotypes of WT M0 BMDMs and those polarized to M1 and TCM states were characterized using flow cytometry. WT M0 BMDMs expressed low levels of costimulatory factor CD80, inducible nitric oxide synthase (iNOS), macrophage mannose receptor (CD206), and arginase-1 (Arg1) (**Figure 1B**). WT M1 BMDMs highly upregulated CD80 and iNOS, whereas WT TCM BMDMs significantly upregulated CD206 and ARG1 (**Figure 1B**). WT TCM BMDMs also upregulated CD80 (**Figure 1B**). Given these marker level shifts with polarization, CD80 and iNOS were used to define M1 polarization, while CD206 and Arg1 were used to define TCM polarization (3, 33, 34). The baseline polarization profile of WT BMDMs treated with isotype antibodies was also confirmed by flow cytometry and largely matched the profile of WT BMDMs (35) (**Supplementary Figure 1C**).

The expression of polarization markers was compared between WT and KO BMDMs and isotype and PD-L1 blockade treated BMDMs in M0, M1, and TCM states. M0 macrophages consistently had low surface levels of the markers measured, resulting in no significant differences in the M1 markers CD80 and iNOS (**Supplementary Figure 1D**), or TCM markers CD206 and Arg1 (**Supplementary Figure 1E**). Both PD-L1 KO and PD-L1 blockade M1-polarized BMDMs had significantly decreased CD80 surface levels as compared to WT and isotype treated M1-polarized BMDMs (**Figure 1C**). While there was no change in the M1 marker iNOS in PD-L1 KO BMDMs, there was an increase in iNOS expression upon PD-L1 blockade (**Figure 1C**). TCM markers CD206 and Arg1 were largely unchanged in M1-polarized KO BMDMs compared to WT, though there was a statistically significant decrease in CD206 surface levels upon PD-L1 blockade in M1-polarized macrophages (**Figure 1D**). To provide biological context for the decreased CD206 expression within M1-polarized macrophages, CD206 and Arg1 expression levels were graphed alongside WT TCM macrophage controls. Likewise, WT M1 levels of M1 markers CD80 and iNOS were graphed as controls for TCMs. PD-L1 KO TCM polarized BMDMs showed a trend of a decrease in CD80 surface levels but no change in iNOS expression, while PD-L1 blockade resulted in a significant decrease in CD80 surface levels and no change in iNOS expression (**Figure 1E**). Analysis of TCM markers CD206 and Arg1 in TCM polarized macrophages demonstrated no significant changes upon PD-L1 KO or blockade (**Figure 1F**). Thus far, the most notable impact of PD-L1 KO or blockade was the consistent decrease in CD80 surface levels across conditions.

To confirm that the observed changes in marker levels were not due to differential cell survival across conditions, we analyzed viability of WT, KO, isotype and PD-L1 blockade BMDMs following 48 hours of M1 and TCM polarization. We observed maintained or increased viability with PD-L1 KO or blockade in all conditions. Notably, TCM-polarized KO BMDMs and M1-polarized PD-L1 blockade BMDMs demonstrated significantly improved survival of the stress of polarization relative to their controls (34–36) (**Supplementary Figure 2A**). These differences suggest that the limited changes observed were not due to preferential survival of less polarized macrophages.

To compare the effects of PD-L1 KO versus PD-L1 blockade on macrophage polarization, we compared log_2_ fold changes relative to respective controls for markers with statistically significant differences. For M1-polarized macrophages we observed no difference in fold change of levels of CD80 or iNOS between PD-L1 KO and PD-L1 blockade macrophages (**Figure 1G**). While there was a significant difference between KO and anti-PD-L1 treated M1 macrophages in CD206 and Arg1 expression, the biological relevance of these changes was negligible when compared to TCM controls (**Supplementary Figure 2B**). When comparing PD-L1 KO and PD-L1 blockade TCM-polarized macrophages, no significant differences in fold change were observed in CD80 (**Figure 1H**), iNOS, CD206, or Arg1 expression (**Supplementary Figure 2C**). Thus, based on canonical inflammatory and tumor-conditioned markers, we did not observe broad differences in macrophage polarization caused by genetic deletion or antibody-mediated blockade of PD-L1 *in vitro*, especially compared with bona fide M1 or TCM marker levels. However, the decrease in CD80 surface levels upon either PD-L1 KO and blockade was consistent.

### PD-L1 blockade during and after macrophage polarization produces comparable effects

We tested the effects of PD-L1 blockade on maintenance of M1 and TCM polarization states by comparing M1 and TCM BMDMs treated with isotype or anti-PD-L1 antibody during a 96-hour window (**Figure 2A**, top row) with M1 and TCM BMDMs polarized for 48 hours prior to treatment with isotype or PD-L1 blockade for a subsequent 48 hours (**Figure 2A**, bottom row). Efficacy of PD-L1 blockade was confirmed by flow cytometry (**Supplementary Figure 3A**). The baseline isotype treated expression profile for each polarization condition was comparable between the control 96-hour polarization group and the experimental 48-hour treatment post 48-hour polarization group (**Supplementary Figures 3B, C**; “96hr Polarization” compared to “Post-Polarization”). Within M1 BMDMs in both conditions, CD80 surface levels were significantly reduced with anti-PD-L1 while iNOS did not change (**Figure 2B**). Analysis of TCM markers CD206 and Arg1 resulted in a significant decrease in CD206 expression in both conditions, but no significant change in Arg1 expression (**Figure 2C**). There was no significant difference in the log_2_ fold decrease in expression of CD80 and CD206 of PD-L1 blockade over isotype treated M1 BMDMs in both conditions (**Figure 2D**). While there was a statistically significant decrease and increase respectively in CD80 and iNOS levels in the 96-hour polarization group upon PD-L1 blockade, the expression levels were minimal compared to the positive controls (**Supplementary Figures 3D, E**).

**Figure 2:**
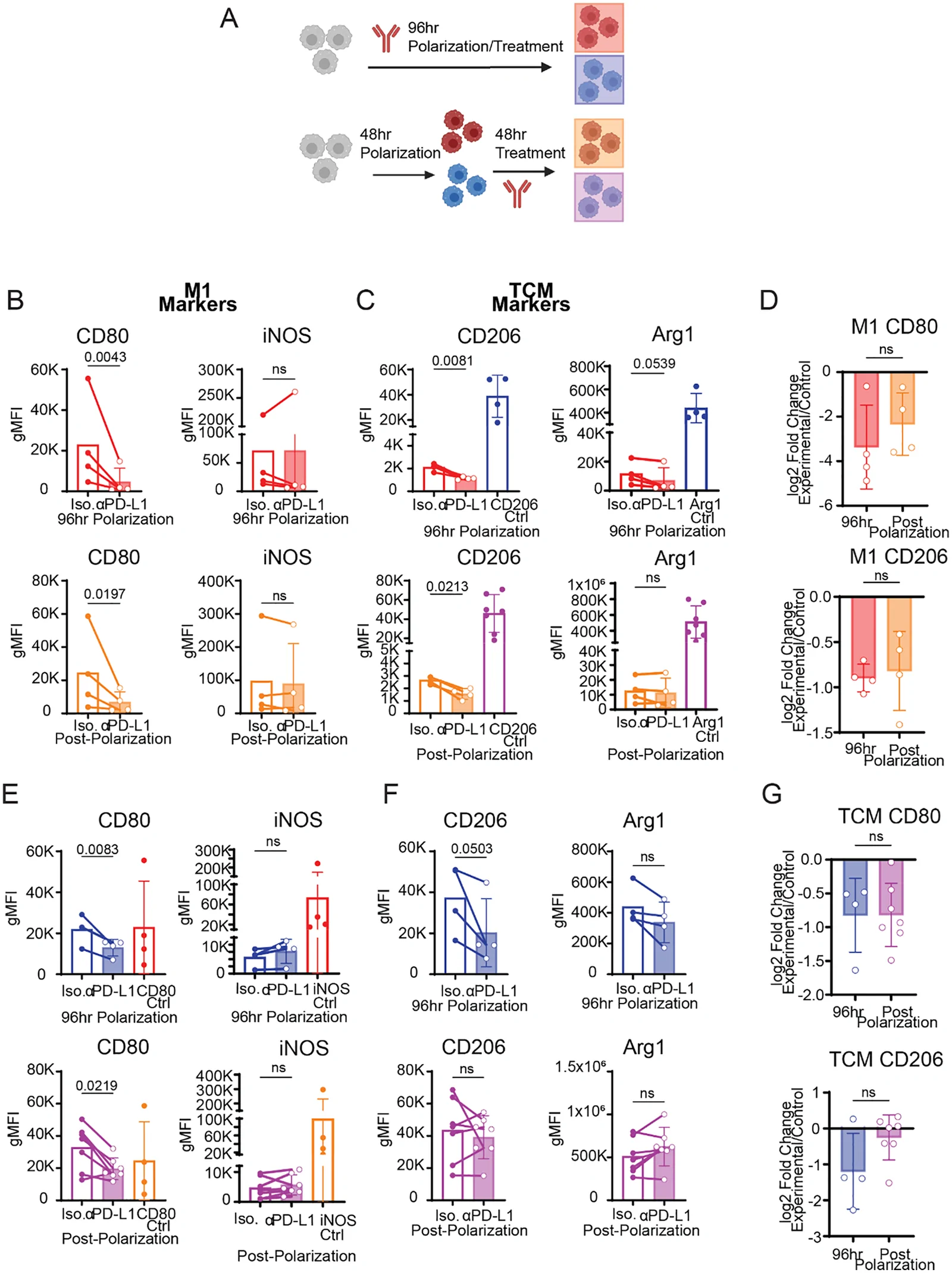
PD-L1 blockade during and after macrophage polarization produces comparable effects. **(A)** Experimental setup: Bone marrow cells isolated from C57BL/6J mice were differentiated into naïve M0 BMDMs using M-CSF, and M1 and TCM polarized by IFNy and LPS or IL-4/IL-10 and E0771 tumor media respectively. Macrophages were polarized for 96hrs. They were split into groups where they were either treated with anti-PD-L1 or Isotype control for 96hrs or allowed to polarize for 48hrs before being treated with antibodies for the second 48hrs. The 96hr polarization/treatment group was color coded Red=M1 and Blue=TCM. The Post-polarization treatment group was color coded Orange=M1 and Purple=TCM. **(B)** M1 marker expression of M1 polarized BMDMs 96hr polarization/treatment and post-polarization treatment (N = 4 mice)(CD80 geometric mean fold change 96hr = 0.14 (95% CI 0.06 – 0.31) post-polarization = 0.31 (95% CI 0.14 – 0.70)). **(C)** TCM marker expression of M1 polarized BMDMs 96hr polarization/treatment and post-polarization treatment (N = 4 mice)(CD206 geometric mean fold change 96hr = 0.54 (95% CI 0.40 – 0.74) post-polarization = 0.57 (95% CI 0.38 – 0.85)). **(D)** Log2 fold change of CD80 and CD206 expression comparing 96hr polarization/treatment with post-polarization treatment (N = 4 mice). **(E)** M1 marker expression of TCM polarized BMDMs 96hr polarization/treatment and post-polarization treatment (96-hour polarization N = 4 mice post-polarization N = 7)(CD80 geometric mean fold change 96hr = 0.59 (95% CI 0.45 – 0.77) post-polarization = 0.63 (95% CI 0.44 – 0.91)). **(F)** TCM marker expression of TCM polarized BMDMs 96hr polarization/treatment and post-polarization treatment (96-hour polarization N = 4 mice post-polarization N = 7) (CD206 geometric mean fold change 96hr = 0.48 (95% CI 0.23 – 1)). **(G)** Log2 fold change of CD80 and CD206 expression comparing 96hr polarization/treatment with post-polarization treatment (96-hour polarization N = 4 mice post-polarization N = 7). Each dot represents a biological replicate. Each biological replicate represents the mean of a technical triplicate derived from one mouse. All conditions were repeated on at least 2 experimental days. Data analysis by two-tailed paired T test of log10 transformed data. Log2 fold change of Blockade over Isotype in 96hr and Post-Polarization groups performed per paired mouse and analyzed with two-tailed Welch’s corrected T test. Constants 1499 and 2413 were used for M1 96hr and post-polarization CD206 samples. Constants 2196 and 2243.45 were used for TCM 96hr and post-polarization iNOS samples.

Within TCM-polarized BMDMs, the M1 marker CD80 was again significantly decreased in both PD-L1 blockade treatment during polarization and post-polarization while iNOS was unchanged (**Figure 2E**). The TCM marker CD206 trended toward a decrease in the 96-hour polarization treatment condition but not in the post-polarization condition, while Arg1 was unchanged in both (**Figure 2F**). When we compared the log_2_ fold changes in expression of CD80 and CD206 levels, we observed no difference in CD80 surface level fold changes between treatment conditions and a trend toward a larger decrease in CD206 surface levels during the 96-hour polarization condition (**Figure 2G**). Taken together, changes in polarization marker expression upon PD-L1 blockade post-polarization largely matched the changes observed upon treatment during polarization. Thus, we continued our analyses using PD-L1 blockade during polarization.

### Macrophage phagocytosis is unaffected by interference of PD-L1

We utilized pH-sensitive phRodo zymosan particles to determine general phagocytic capacity by imaging particle engulfment by BMDMs *in vitro* using the Incucyte live-cell imaging platform. Phagocytic rate was quantified as the area of phRodo fluorescence normalized to cell phase area over incubation time, with the area under the curve (AUC) calculated for WT versus PD-L1 KO (**Figures 3A–C**), and isotype versus PD-L1 blockade (**Figures 3D–F**) BMDMs in M0, M1, and TCM states. After 3 hours in culture, cells were harvested to quantify the total cell numbers and the frequencies of engulfed particles at the endpoint by flow cytometry. There were no significant changes to the phagocytic capacities of M0, M1, or TCM BMDMs in PD-L1 KO macrophages, as measured by Incucyte or by flow cytometric analysis (**Figures 3A–C**). There was also no change in phagocytic capacities of M0 and M1 BMDMs upon PD-L1 blockade (**Figures 3D,E**). While there was no change in particle engulfment by TCM BMDMs treated with PD-L1 blockade, there was a modest decrease in the percentage of phagocytic macrophages collected and analyzed by flow cytometry at the 3-hour timepoint (**Figure 3F**). Thus, functional macrophage activation, as reflected by zymosan engulfment, was not regulated by PD-L1 KO or blockade under the conditions tested.

**Figure 3:**
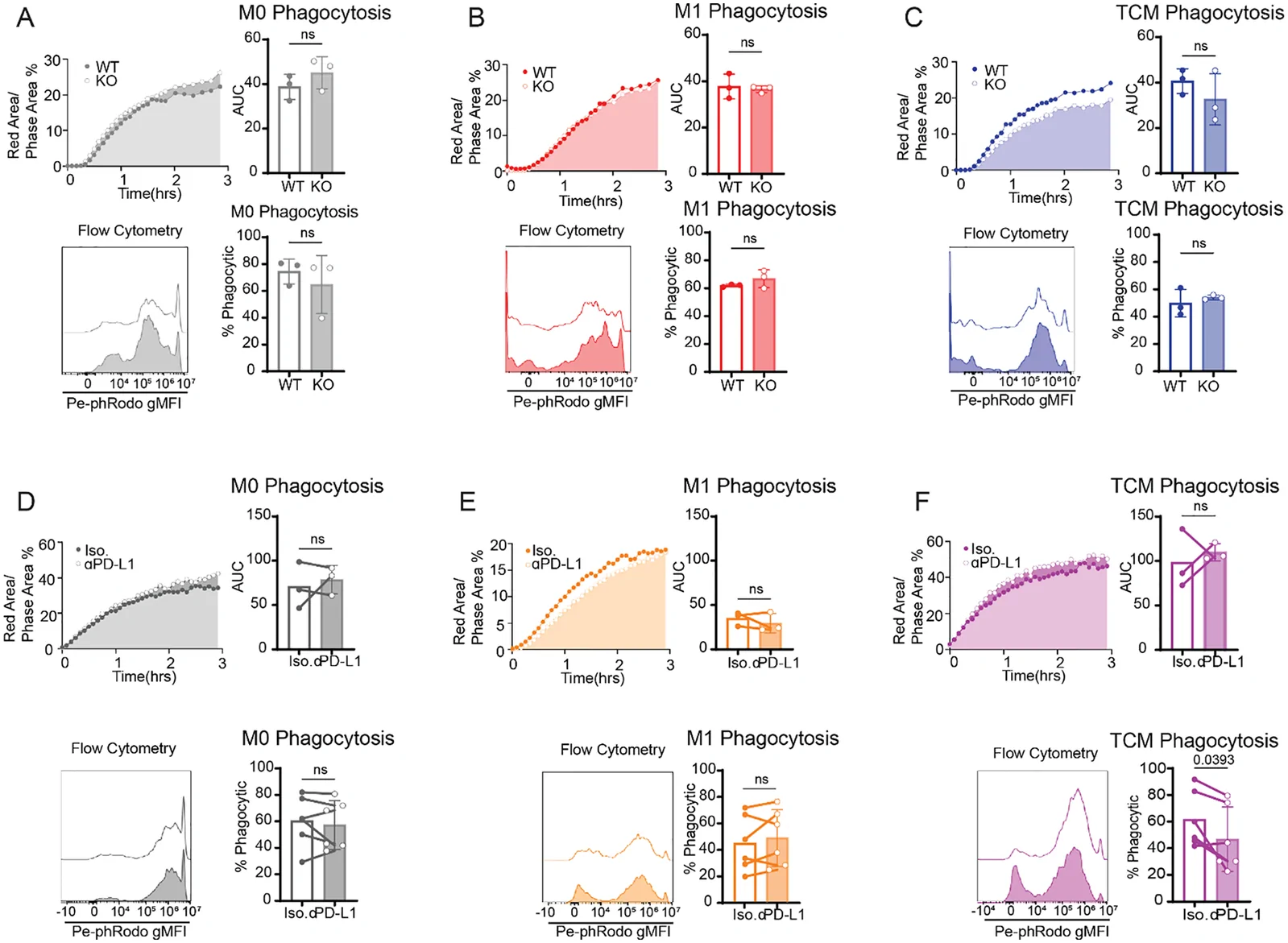
Macrophage phagocytosis is unaffected by interference of PD-L1. WT and KO BMDMs were incubated with pHrodo Red labeled zymosan particles and particle engulfment over time was measured, as defined by percentage of pHrodo-positive Red cell area normalized to total cell phase area. **(A)** Naïve BMDMs WT vs KO 3hr incubation summarized Red Area/Phase Area curves with corresponding analysis of the area under the curve (AUC) and flow cytometry analysis of red fluorescent BMDMs after the 3hr incubation. (N = 3 Mice, each replicate a mean of triplicate wells). **(B)** M1 BMDMs WT vs KO AUC and flow analysis. (N = 3 Mice, each replicate a mean of triplicate wells). **(C)** TCM WT vs KO AUC and flow analysis. (N = 3 Mice, each replicate a mean of triplicate wells). **(D)** Naive Isotype vs anti-PD-L1 AUC and flow analysis. (AUC N = 3 Mice, each replicate a mean of 2–3 wells, Flow analysis N = 6 mice). **(E)** M1 Isotype vs anti-PD-L1 AUC and flow analysis. (N = 3 Mice, each replicate a mean of 2–3 wells, Flow analysis N = 6 mice). **(F)** TCM Isotype vs anti-PD-L1 AUC and flow analysis. (N = 3 Mice, each replicate a mean of 2–3 wells, Flow analysis N = 6 mice). Each dot represents a biological replicate. Each biological replicate represents the mean of a technical triplicate derived from one mouse. Isotype vs Blockade includes 2 experimental days with flow analysis. Data analysis performed with two-tailed Welch’s T test and paired T test for WT vs KO and Isotype vs anti-PD-L1 respectively.

### PD-L1 blockade and knockout reduce surface CD80 and differentially alter immune regulatory molecules

Given the consistent reduction in CD80 surface levels across conditions, we performed orthogonal experiments to investigate this finding. We first compared CD80 surface level stains with combined surface and intracellular, or total, staining upon PD-L1 blockade in both M1 and TCM BMDMs. Interestingly, while both the isotype and PD-L1 blockade groups increased upon total staining, the significant decrease between isotype and PD-L1 blockade was similar between surface and total staining (**Supplementary Figure 4A**). Additionally, analysis of RNA transcript levels with qPCR demonstrated no difference between isotype and PD-L1 blockade or WT and KO in M1 and TCM BMDMs (**Supplementary Figure 4B**), suggesting regulation at the protein level. Due to the close relationship between CD80 and PD-L1, we also investigated PD-L1 protein and RNA levels upon blockade and found that an intracellular pool of PD-L1 was untouched by blockade and RNA transcripts were unchanged (**Supplementary Figure 4C**).

As CD80 is a costimulatory factor, we expanded analysis to include antigen presentation molecules: major histocompatibility complexes class I (MHCI) and class II (MHCII), and immune checkpoint molecules: PD-1 and programmed cell death-ligand 2 (PD-L2). Similar to what was observed with polarization-associated molecules (CD80, iNOS, CD206, Arg1), WT M0 BMDMs and isotype control-treated BMDMs had low surface levels of MHCI, MHCII, PD-1, PD-L1, and PD-L2 (**Supplementary Figure 5**). Upon M1 polarization, WT BMDMs upregulated surface levels of MHCI, MHCII, and PD-L1, whereas WT TCM BMDMs upregulated MHCI, MHCII, PD-1, PD-L1, and PD-L2 (**Supplementary Figure 5A**). Isotype-treated WT BMDMs recapitulated these changes, with the exception of MHCII, in which TCMs expressed greater surface levels than M1 (**Supplementary Figure 5B**). Thus, M1 and TCM BMDMs have unique signatures with respect to antigen presentation machinery. WT and PD-L1 KO M0 BMDMs had similarly low surface levels of MHCI, MHCII, PD-1, and PD-L2 (**Supplementary Figure 5C**). PD-L1 blockade of M0 polarized BMDMs also did not impact surface levels of MHCI, MHCII, PD-1, or PD-L2 (**Supplementary Figure 5D**).

There were no changes to the surface levels of MHCI and MHCII between WT and PD-L1 KO or isotype and PD-L1 blockade M1 BMDMs (**Figure 4A**). PD-L1 KO TCMs showed significantly increased MHCII surface levels, approaching levels of MHCII-positive controls, whereas PD-L1 blockade did not significantly affect MHCII surface levels compared to the isotype control (**Figure 4B**). In line with these observations, there was no difference in log_2_ fold changes in response to PD-L1 KO or blockade in M1 or TCM BMDMs in levels of MHCI or PD-L1 (**Supplementary Figures 6A, B**). In contrast, comparing log_2_ fold changes in marker surface levels between PD-L1 KO and blockade revealed no difference in M1 BMDMs (**Figure 4C**), but an increase in MHCII levels upon KO compared to PD-L1 blockade in TCM BMDMs (**Figure 4D**).

**Figure 4:**
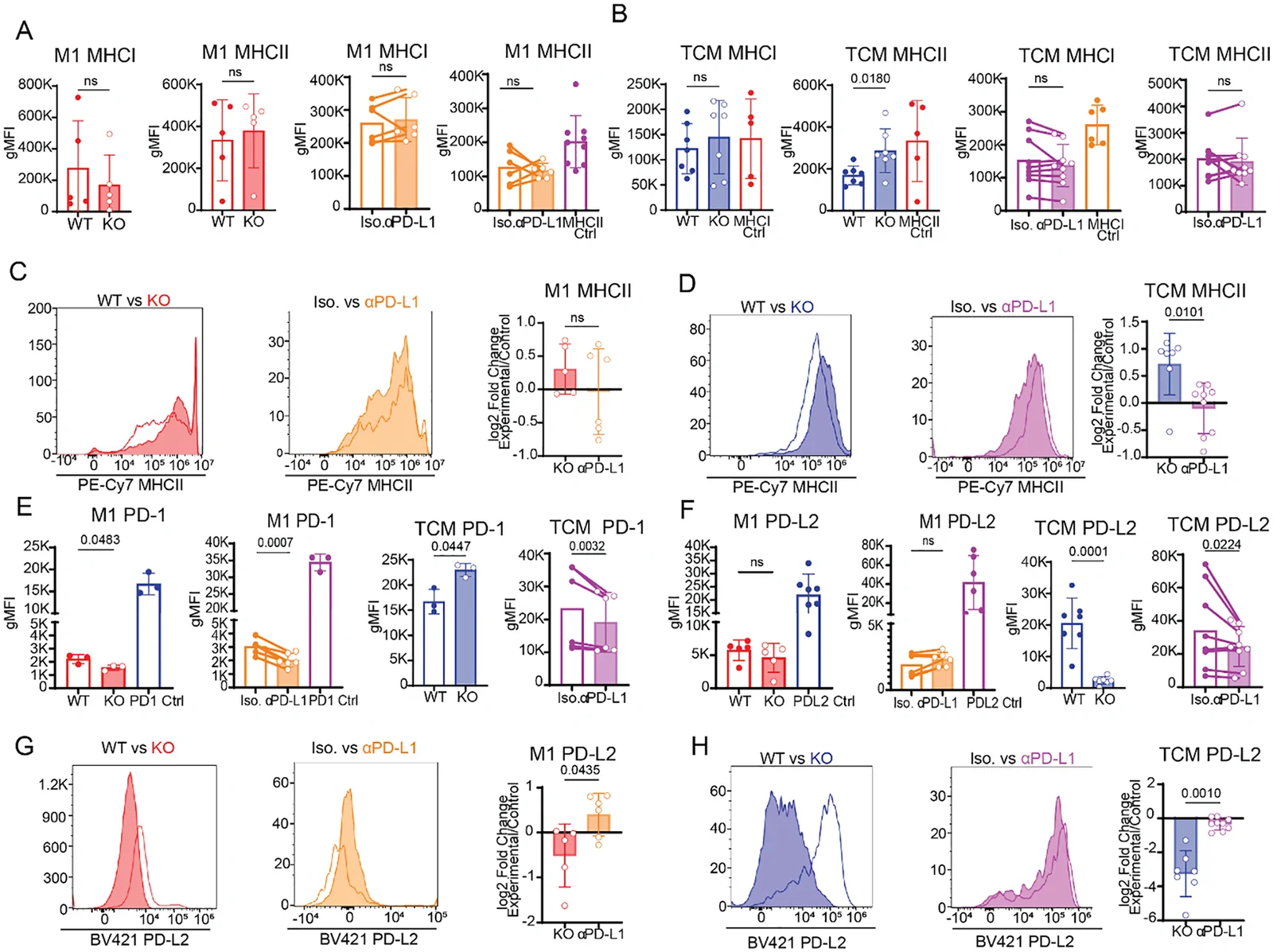
PD-L1 blockade and knockout differentially regulate antigen presentation and checkpoint molecules. **(A)** Antigen presentation molecules MHCI and MHCII expression in M1 polarized BMDMs comparing WT vs KO and Iso vs Blockade. (WT vs KO N = 5 mice, Iso vs Blockade N = 6 mice). **(B)** Antigen presentation molecules MHCI and MHCII expression in TCM polarized BMDMs comparing WT vs KO and Iso vs Blockade. (WT vs KO N = 7 mice, Iso vs Blockade N = 9 mice) KO Geometric mean fold changes: MHCII = 1.64 (95% CI 1.11 – 2.43). **(C)** Log2 fold change of KO/WT and blockade/iso of MHCII expression in M1 polarized BMDMs. (KO N = 5 mice, Blockade N = 6 mice). **(D)** Log2 fold change of KO/WT and blockade/iso of MHCII expression in TCM polarized BMDMs. (KO N = 7 mice, Blockade N = 9 mice). **(E)** Checkpoint marker PD-1 in M1 and TCM polarized BMDMs comparing WT vs KO and Iso vs Blockade. (WT vs KO M1 N = 3 mice, WT vs KO TCM N = 5 mice, Iso vs Blockade N = 6 mice) KO M1 PD-1 geometric mean fold change = 0.7 (95% CI 0.49-0.99), TCM PD1 = 1.38 (95% CI 1.02 – 1.89), Blockade M1 Geometric mean fold change PD-1 = 0.66 (95% CI 0.57 – 0.76), TCM PD-1 = 0.85 (95% CI 0.78 – 0.92). **(F)** Checkpoint marker PD-L2 in M1 and TCM polarized BMDMs comparing WT vs KO and Iso vs Blockade. (M1 N = 5 mice, TCM WT vs KO N = 7 mice, TCM Iso vs Blockade N = 9 mice). KO Geometric mean fold change: PDL2 = 0.11 (95% CI 0.05 – 0.24). Blockade Geometric mean fold change: PDL2 = 0.79 (95% CI 0.66 – 0.96). **(G)** Log2 fold change of KO/WT and blockade/iso of PD-L2 expression in M1 polarized BMDMs. (KO N = 5 mice Blockade N = 6 mice). **(H)** Log2 fold change of KO/WT and blockade/iso of PD-L2 expression in TCM polarized BMDMs. (KO N = 7 mice Blockade N = 9). Each dot represents a biological replicate. Each biological replicate represents the mean of a technical triplicate derived from one mouse. All conditions were repeated on at least 2 experimental days with the exception of PD-1 in the WT vs KO experiments. Analysis performed with two-tailed Welch’s T test on log10 transformed data for WT vs KO groups. Two-tailed Paired T tests on log10 transformed data were used for Isotype vs Blockade groups. For both, constants to ensure positive values were used. Constants 1321 and 2021.67 for M1 PD-L2 WT/KO and Iso/aPD-L1 respectively. Two-tailed Welch’s T tests were performed on log2 fold change of experimental condition (KO, anti-PD-L1) to control condition (WT, Isotype) for PD-L1 deficiency and blockade BMDMs.

Analysis of the checkpoint markers PD-1 and PD-L2, which are predominantly expressed in the TCM condition (**Supplementary Figure 5A**), revealed a statistically significant decrease in PD-1 surface levels in PD-L1 KO M1 BMDMs as compared to WT and in PD-L1 blockade treated M1 BMDMs compared to isotype (**Figure 4E**). However, the biological significance of these changes in M1 BMDMs may be limited when compared to the 8-fold to 10-fold higher surface level expression of PD-1 by TCM controls. Analysis of TCM BMDMs indicated that PD-L1 KO BMDMs exhibited increased PD-1 surface levels while PD-L1 blockade decreased PD-1 surface levels (**Figure 4E**). As both PD-L1 deletion and blockade in M1 BMDMs were associated with reduced PD-1, we conducted flow cytometric analyses of surface versus total PD-1, in which the total PD-1 detection revealed no change while the surface PD-1 detection was decreased in blockade (**Supplementary Figure 6C**). Levels of *Pdcd1* transcripts were significantly elevated in blockade (**Supplementary Figure 6C**). To support our findings of PD-1 expression by BMDMs, we further examined PD-1 with full minus one (FMO) controls in M1 and TCM BMDMs (**Supplementary Figure 6D**). Comparing log2 fold changes in PD-1 surface levels in M1 and TCM BMDMs, PD-L1 KO and PD-L1 blockade exhibited comparable decreases in expression in M1 BMDMs. PD-L1 KO in TCM BMDMs resulted in an increase in PD-1 surface levels, while PD-L1 blockade resulted in a decrease of surface levels (**Supplementary Figure 6E**).

We observed slight, though not statistically significant changes to PD-L2 in M1 BMDMs with blockade and deletion, though PD-L2 expression was generally low in M1 BMDMs (**Figure 4F**). In the TCM condition, PD-L2 surface levels were significantly decreased upon PD-L1 blockade as compared to isotype, and were almost undetectable in PD-L1 KO (**Figure 4F**). Along these lines, PD-L2 surface levels were also differentially impacted, with an decrease in PD-L1 KO M1 BMDMs as compared to WT, and an increase in PD-L1 blockade M1 BMDMs as compared to isotype (**Figure 4G**). While PD-L2 levels were decreased in both PD-L1 KO and blockade groups in TCM BMDMs, the magnitude of the decrease was significantly greater in PD-L1 KO TCM compared to WT than the PD-L1 blockade TCM compared to isotype control (**Figure 4H**). Given reduction of PD-L2 in TCM BMDMs with both PD-L1 deletion and blockade, we compared surface and total staining of PD-L2 in isotype versus blockade treated TCM BMDMs (**Supplementary Figure 6F**). PD-L2 surface and total detection were comparable between isotype and blockade treated TCM BMDMs, as were levels of RNA transcript (**Supplementary Figure 6F**). Together, these results indicate that PD-L1 intrinsically regulates CD80 protein levels and influences other immune regulatory surface molecule levels on macrophages.

## Discussion

We investigated the cell-intrinsic role of PD-L1 in macrophages by comparing genetic deletion and antibody-mediated blockade with PD-L1-sufficient controls across multiple polarization states. Our findings suggest a macrophage intrinsic role of PD-L1 that influences the surface levels of immunomodulatory molecules. Notably, both genetic deletion and antibody-mediated blockade of PD-L1 consistently reduced surface CD80 expression, suggesting that PD-L1 contributes to the maintenance of CD80 surface expression in macrophages.

Contrary to reports that PD-L1 blockade promotes inflammatory macrophage polarization, we observed minimal effects on classical polarization markers. Previous models indicated that PD-L1 blockade increased pro-inflammatory features MHCI, MHCII, and CD86 and decreased Arg1 by comparing fold-change of marker expression levels within macrophages treated with PD-L1 blockade and isotype controls (27, 36). While these studies reported statistically significant changes in marker expression, the biological relevance of these results was less clear. In our analysis we compared the changes in marker expression with WT M1- or TCM-polarized macrophages as controls to provide context for the relative changes in polarization profiles. We also analyzed naïve, M1- and TCM-polarized macrophages, in which we demonstrated varied responses to PD-L1 interference. To ensure that the lack of changes in polarization was not due to preferential survival of less polarized macrophages upon polarization, we compared cell viability between WT, KO, isotype and antibody treated groups in each polarization condition and observed no decrease in viability (**Supplementary Figure 2**). Taken together, our findings suggest that PD-L1 perturbation is insufficient to broadly regulate macrophage polarization in isolated BMDMs. While perturbations to PD-L1 may induce statistically significant fold changes in polarization marker expression levels, they may not be so relevant when compared to that of bona fide M1 or TCM-polarized macrophages. However, this conclusion should be interpreted within the context of our *in vitro* system, as PD-L1 may exert more pronounced effects within tissue microenvironments where macrophages are exposed to complex cellular and inflammatory signals.

Although macrophage activation is frequently accompanied by changes in phagocytic function, neither genetic deletion nor antibody blockade of PD-L1 altered zymosan bead uptake in our system. Phagocytosis is a canonical function of macrophages that is frequently associated with macrophage activation. Prior studies have reported increased phagocytic function of macrophages following interference with the PD-1/PD-L1 axis in several tumor models, whereas *in vitro* monoculture systems have reported variable results (24, 25, 36, 37). In our reductionist *in vitro* monoculture, neither genetic deletion nor antibody blockade of PD-L1 substantially altered zymosan particle uptake across polarization conditions. This finding is consistent with our observation that PD-L1 perturbation did not broadly alter macrophage polarization and suggests that the enhanced phagocytosis observed *in vivo* may depend on additional cellular and inflammatory cues in the tumor microenvironment rather than PD-L1 perturbation alone.

A consistent change to macrophage phenotype upon PD-L1 deletion or blockade was a reduction in CD80 surface levels, which we had also previously observed in our melanoma model (25). CD80 and PD-L1 have been shown to bind in cis on the plasma membrane of dendritic cells (38–40). Consistent with our findings, Zhao et al. (39) reported reduced CD80 surface levels following PD-L1 blockade and attributed this effect to CTLA4-mediated trans-endocytosis of CD80. However, because our experiments were performed in BMDM monocultures lacking CTLA4-expressing T cells, the consistent reduction in CD80 surface and total protein levels observed following PD-L1 blockade and the surface reduction upon genetic deletion cannot readily be explained by this mechanism. With the evidence that PD-L1 blockade does not change CD80 transcripts, our findings suggest that PD-L1 contributes to the maintenance of CD80 surface levels through a macrophage-intrinsic mechanism, potentially involving regulation of CD80 stability, trafficking, or surface retention. While the lack of a change in CD80 transcript levels upon PD-L1 blockade could be due to temporal regulation of protein levels, the evidence that PD-L1 knockout also reduced surface and total CD80 protein expression without changing transcript levels strengthens the model of PD-L1 mediated protein level regulation of CD80.

The observed reduction in PD-L2 was particularly unexpected. Although direct interactions between PD-L1 and PD-L2 have not been described, both ligands bind PD-1 and share overlapping immunoregulatory functions. PD-L2 expression is typically lower than PD-L1, but exhibits approximately three-fold greater affinity for PD-1 (41). In our model, PD-L1 was highly expressed in M1 macrophages and moderately expressed in TCMs, whereas PD-L2 surface expression was largely restricted to the TCM state (**Supplementary Figure 4**). Given these overlapping functions, we anticipated that loss of PD-L1 signaling might be accompanied by compensatory upregulation of PD-L2. Instead, PD-L2 surface levels decreased significantly following PD-L1 blockade and were nearly abolished in PD-L1-deficient macrophages (**Figure 4**). These findings suggest that PD-L1 and PD-L2 may be coordinately regulated rather than functionally compensatory, raising the possibility that PD-L1 contributes to the maintenance of PD-L2 surface levels. Further studies are needed to define the mechanisms underlying this relationship.

When comparing changes in MHCII surface levels, we observed distinct effects of PD-L1 genetic deletion and antibody-mediated blockade in TCM BMDMs, with MHCII surface levels increasing following PD-L1 knockout but trending down following PD-L1 blockade (**Figure 4**). Similarly, PD-1 surface levels differentially shifted with an increase upon PD-L1 knockout and a decrease upon PD-L1 blockade in TCM BMDMs (**Figure 4**). This divergence is notable given the concurrent alterations in other molecules involved in T-cell communication, including CD80 and PD-L2. Together, our data indicated that genetic deletion and antibody-mediated blockade of PD-L1 do not produce equivalent effects on macrophage phenotype.

The distinct changes between PD-L1 knockout and blockade could be due to changes in macrophage maturation in the PD-L1 knockout model that could not be replicated in the blockade model. Though, our analysis of PD-L1 did demonstrate biologically distinct profiles of PD-L1 knockout and blockade macrophages with blockade retaining an intracellular pool and RNA expression of PD-L1 (**Supplementary Figures 1A–C**, 4C). Additionally, the consistent reduction of CD80 protein levels with no change in RNA transcript levels despite the biologically distinct nature of PD-L1 knockout and blockade strengthens the evidence of the importance of PD-L1 in CD80 regulation.

Although the specific effects of PD-L1 deletion and blockade differed across molecules, the most substantial changes were consistently observed in immune regulatory protein expression. This is a surprising result when considering the prior literature suggested the largest changes would be seen in canonical polarization markers. These findings suggest that PD-L1 may have a greater influence on macrophage interactions with and instruction of adaptive immune cells than on canonical polarization profiles. One possible explanation for the disparity between our findings and previous studies is that PD-L1 dependent changes in immune regulatory molecules might alter macrophage interactions with nearby immune cells. These altered interactions could subsequently provide paracrine signals that influence changes in macrophage polarization and phagocytic capacity. In this model, macrophage-intrinsic PD-L1 functions would influence macrophage communication with the immune microenvironment, potentially exerting broader effects on macrophage activation only after reciprocal interactions with the tumor microenvironment. Such effects would not be expected in a reductionist monoculture system.

## Conclusions

The importance of macrophage PD-L1 in the tumor microenvironment continues to expand as the functions of both macrophages and PD-L1 are increasingly appreciated. Here, we demonstrate that genetic deletion and antibody-mediated blockade of PD-L1 reduce CD80 surface expression on macrophages. These findings support a cell-intrinsic role for PD-L1 in macrophages that extend beyond its canonical function as a ligand for PD-1 on T cells. Further investigation of the mechanisms by which PD-L1 regulates macrophage function may provide new opportunities to modulate the tumor microenvironment and improve the efficacy of immunotherapeutic strategies.

### Limitations

Our model was refined to *in vitro* assays utilizing one Tumor Conditioned Media (EO771) and only female mice due to the nature of triple negative breast cancer. This work overlapped with some methodology done in previous work in the lab (Kwok et al., 2025) using the B16 melanoma model; however, some results differed. This difference suggests cancer-specific regulation of macrophage function. As mentioned above, because the PD-L1 KO model used is missing PD-L1 expression throughout development and maturation of monocytes to macrophages, there could be differences in macrophage maturation that would not be caused by blockade after differentiation. We controlled for differences in polarization that might occur due to PD-L1 loss by treating with PD-L1 blockade during polarization, but a control for differentiation from monocytes to macrophages could be achieved by blockade during M-CSF mediated differentiation. Reintroduction of PD-L1 constructs could also help parse out any differences due to PD-L1 loss before differentiation. To further investigate PD-L1 functions in macrophages, the ability to reintroduce PD-L1 expression in PD-L1 knockout backgrounds would strengthen these analyses. Macrophage phagocytosis is regulated by diverse pathways which require multiple methods of analysis to clarify. Here we analyzed the effects of PD-L1 interference on a single phagocytic pathway. Our analysis does not include the effects of PD-L1 on phagocytosis of opsonized targets or apoptotic cells, but points to a general fitness in a core macrophage function.

### Future directions

The evidence we have gathered suggests that direct contact between PD-L1 and CD80 is important for maintaining CD80 expression levels on the cell surface. Previous studies have demonstrated the importance of PD-L1 and CD80 cis-interactions for dendritic cell migration and that the intracellular tail of PD-L1 is important for this interaction (39). However, it has not been clarified whether PD-L1 simply stabilizes CD80 on the cell surface or plays a role in its stabilization and trafficking to the cell surface. Further studies are needed to clarify this interaction. The loss of PD-L2 surface expression suggests a positive regulation of PD-L2 by PD-L1 expression. Due to the proximity of the PD-L2 gene to PD-L1, mechanisms of coregulation could contribute to the loss of PD-L2 upon PD-L1 knockout, but the decrease in PD-L2 upon PD-L1 blockade reduces the likelihood of that explanation. Investigating potential interactions of PD-L1 and upstream regulators of PD-L2 could clarify this dynamic.

## Materials and methods

### Mice/Cell culture

Wildtype C57Bl/6J mice were purchased from The Jackson Laboratories and PD-L1^-/-^ C57Bl/6 mice were generated and bred in-house (30). Mice were bred and maintained under specific-pathogen free conditions with controlled temperature and 12-hour light/dark day cycle in the Mayo Clinic animal facility. Female mice 6–10 weeks of age were used in experiments in accordance with protocols approved by the Mayo Clinic Institutional Animal Care and Use Committee (AUP #A00005857-21-R24).

E0771 murine triple negative breast cancer cells (ATCC #CRL-3461) were cultured under normoxic conditions at 37 °C, 5% CO_2_ in Dulbecco’s Modified Eagle’s Medium (DMEM; Thermofisher), 10% fetal bovine serum (FBS; Thermofisher).

Naïve, M1, and TCM macrophages were treated with anti-PD-L1 (Clone 10F.9G2 BioXcell BE0101) or isotype matched (IgG2b LTF-2 BioXcell BE0090) at 20ug/mL during polarization (48hrs or 96hrs) and after polarization (48hrs) depending on experimental conditions.

### Generation of bone-marrow derived macrophages

BMDMs were generated using a modified version of the protocol described by (25, 30). Bone marrow from the femur and tibiae of C57Bl/6J or PDL1^-/-^ C57Bl/6J mice were flushed out with serum free DMEM and cultured at densities between 2x10^6 and 4x10^6 per mL on 10cm, 6-well, or 12-well plates in DMEM supplemented with FBS (10%), Penicillin-Streptomycin (1%), and 20 ng/ml of M-CSF (Peprotech) for 6 days. Fresh media was replaced every 3 days. M0 macrophages were cultured in DMEM supplemented with M-CSF (20 ng/ml). Tumor-conditioned macrophages (TCMs) were generated by culturing macrophages in DMEM supplemented with M-CSF (20 ng/ml) and 20 ng/ml of IL-4 and IL-10 (Peprotech), and E0771 tumor conditioned media. M1 macrophages were generated by culturing macrophages in DMEM supplemented with M-CSF (20 ng/ml, Peprotech), 10 ng/ml of LPS (Sigma Aldrich), and 20 ng/ml of IFNγ (Peprotech). Macrophages were polarized for 48 hours then detached with Versene (Thermofisher), washed with PBS, then used for downstream applications such as flow cytometry, western blots, and imaging.

### Flow cytometry

*In vitro* cultures were washed with warm PBS, then detached from plates with Versene (Thermofisher) and washed with FWB (1x PBS + 2% fetal bovine serum). Cells were stained with fluorochrome-conjugated antibodies for 20min at 4 °C, then washed with FWB. For intracellular staining, cells were fixed for 20 min at 4 °C using the FOXP3 Fixation/Permeabilization kit (Thermofisher) as per manufacturer’s instructions then washed in permeabilization buffer. Cells were then stained with fluorochrome-conjugated intracellular antibodies for 20 min at 4 °C, washed, and resuspended in FWB. Samples were read on a Cytek Aurora cytometer and data was analyzed using FlowJo software (v10, Treestar).

**Table 1:** List of conjugated antibodies and dyes used for flow cytometry assays.

Antibodies	Source	Identifier
CD80 – BUV615(Clone 16-10A1)	BD Biosciences	Cat#751328
iNOS – AF594(Clone W16030C)	Biolegend	Cat#696804
CD206 – BV605(Clone C068C2)	Biolegend	Cat#141721
Arg1 – PE(Clone W210471)	Biolegend	Cat#165804
MHCI – APC(Clone AF6-88.5.5.3)	Invitrogen	Cat#17-5958-82
MHCII – PE/Cy7(Clone M5/114.15.2)	Biolegend	Cat#107630
PD-1 – FITC(Clone 29F.1A12)	Biolegend	Cat#135213
PD-L1 – BUV737(Clone MIH5)	BD Biosciences	Cat#568361
PD-L2 – BV421(Clone Ty25)	Biolegend	Cat#107219
Ki67 – FITC(Clone 11F6)	Biolegend	Cat#151212
DAPI	Thermofisher	Cat#D1306
Zombie Red	Biolegend	Cat#423110
GhostDye UV450	Tonbo Bioscience	Cat#13-0868-T100

### Phagocytosis assays

pHrodo assays were performed with a modified protocol based on (25, 42). BMDMs were polarized to M1 and TCM states for 24 hours, then washed and detached from wells using pre-warmed Trypsin EDTA for 5–10 minutes. Cells were counted and 5 X 10^4^ to 7.5 X 10^4^ were seeded per well into a 96-well flat bottom plate and polarization media returned to wells. After 24 more hours of incubation, 2.5ug of red Zymosan pHrodo bioparticles (Sartorius) were added to each well and placed in an Incucyte incubator (Sartorius), scanned every 5–10 minutes for 3 hours at 10x magnification. After the 3 hour analysis with the incucyte, cells were washed and detached for flow cytometry analysis.

### Cell cycle/viability

2.5 x 10^6^ Bone marrow cells were plated and differentiated in 6-well plates. Once differentiated, they were polarized to M1 and TCM states and WT BMDMs treated with anti-PD-L1 antibody or Isotype controls. Cells were detached using trypsin for 5–10 minutes and stained for viability. They were then fixed using the FOXP3 Fixation/Permeabilization kit and stained for Ki67 for 20min at 4 °C. They were then washed and stained with DAPI for 3–5 minutes at room temperature. They were then analyzed using a BD Symphony cytometer.

### Western blot

Whole cell lysates were prepared in RIPA buffer containing Halt protease inhibitors. Equal amounts of protein (30ug) were separated by SDS-PAGE, transferred to low fluorescence PVDF membranes using a turbotransfer system, and probed with antibodies against PD-L1 (MIH5 eBioscience 14-5982-82) and GAPDH(14C10 Cell Signaling 2118). Blots were visualized using IRDye conjugated secondary antibodies using a LI-COR Odyssey Clx imaging system.

### qPCR

For qPCR analysis WT, KO, Isotype, and anti-PD-L1 treated M1 and TCM BMDMs were lysed and collected with 250-500uL TRIzolol (15596026 ThermoFisher). RNA was isolated using phenol-chloroform extraction and ethanol precipitation as described in the TRIzol manual. Genomic DNA was removed from samples with the ezDNase kit, and cDNA was synthesized using SuperScript IV reverse transcriptase kit (18091150 ThermoFisher). cDNA was quantified using a Qubit 1X dsDNA High Sensitivity kit (Q33230 Invitrogen) and 1-5ng cDNA template per well was used with PowerTrack SYBR green master mix and ROX dye for qPCR reactions (A46109 Applied Biosystems). The following primers were used at a concentration of 400nM per reaction. PD-L1: 5’-3’ Fwd “ACATGAGGATATTTGCTGGCATT”, Rev “TTCCCAGTACACCACTAACGC”. Pdcd1: Fwd “ACCCTGGTCATTCACTTGGG”, Rev “CATTTGCTCCCTCTGACACTG”. PD-L2: Fwd “TCATTGACCCTCTGAGTCGG”, Rev “GGAAGATCAAAGCGATGGTGC”. CD80: Fwd “TGCTGCTGATTCGTCTTTCAC”, Rev “GAGGAGAGTTGTAACGGCAAG”. B-actin: Fwd “GGCACCACACCTTCTACAATG”, Rev “GTGGTGGTGAAGCTGTAGCC”. Relative quantification of each target gene was measured using the housekeeping gene Beta Actin. Statistical analysis was performed on delta Cq values and represented in relative fold change.

### Statistical analysis

Data in this study are presented as means ± SEM for n = 3–9 representing the number of mice in each assay. Each mouse biological replicate, or mouse, is an average of 2–3 technical replicates. Each experimental condition was repeated at least 2 times with the exception of some PD-1 analysis, phagocytosis assays, the total protein stain, and qPCR. Because we were comparing changes to protein levels within and not across polarization states, polarization controls were used to inform on biological relevance of protein levels and were not included in the analysis. Statistical analysis was performed using GraphPad Prism software and can be found in the corresponding figures and figure legends. Depending on the data, Welch’s T test, Lognormal Welch’s T test, Paired and Ratio paired T tests, one-way ANOVA, two-way ANOVA, mixed effects and lognormal one-way ANOVA were performed. When comparing WT and KO conditions, two-tailed Welch’s T test or lognormal Welch’s T tests were used since all samples were derived from independent mice and across multiple experimental days which introduced variability in signal levels. When comparing Isotype and anti-PD-L1 treated samples, two-tailed Paired and ratio paired T tests were used because the Isotype and anti-PD-L1 treated cells were derived from the same mice. One-way ANOVA, mixed effects and lognormal one-way ANOVA’s were used when comparing PD-L1 reduction across conditions or establishing baseline expression of markers in WT or Isotype conditions. Mixed effects analysis were used when sample numbers did not match up between variables. Two-way ANOVA’s were used to compare the effects of anti-PD-L1 treatment on surface protein and total protein levels. Multiple comparisons *post-hoc* analysis was performed with Tukey’s multiple comparisons for one-way ANOVA’s and mixed effects models while Sidaks corrected multiple comparisons were used for two-way ANOVA’s.

## Data Availability

The original contributions presented in the study are included in the article/**Supplementary Material**. Further inquiries can be directed to the corresponding authors.

## Supplementary material

Supplementary Figure 1 (A) PD-L1 knockout and blockade of M1 BMDMs as confirmation of knockout and complete blockade through flow cytometry. (KO N = 5 mice, Blockade N = 3 mice). Statistical analysis was performed using a Lognormal ordinary one-way ANOVA with Tukey’s multiple comparisons Test. (B) Western blot, and qPCR (right figure) of M1 BMDMs as confirmation of PD-L1 knockout (Western Blot N = 1 mouse, qPCR N = 3 mice). For RNA analysis non-amplified replicates were assigned a raw Cq of 40. Each dot represents an averaged technical duplicate of a single mouse. (C) Baseline expression of polarization panel in Isotype treated BMDMs. CD80 TCM expression appears higher than M1 BMDMs due to experimental day variability resulting in outliers. Experimental day matched samples result in M1 BMDMs with the highest expression (M0 N = 6 mice, M1 N = 3 mice, TCM N = 6 mice). Statistical analysis was performed using Mixed-effects analysis with Tukey’s multiple comparisons. (D) M1 marker expression of naïve BMDMs WT/KO and Iso/aPD-L1. (Blockade N = 6 mice, KO N = 7 mice). Statistical analysis was performed with Welch’s T test analysis of Log10 transformed data for WT/KO analysis and Ratio paired T test for Iso/aPD-L1 analysis. (E) TCM marker expression of naïve BMDMs WT/KO and Iso/aPD-L1 (Blockade N = 6 mice, KO N = 7 mice). The remaining statistical analysis was performed with Welch’s T test analysis of Log10 transformed data for WT/KO analysis and Ratio paired T test for Iso/aPD-L1 analysis.

Supplementary Figure 2 (A) Cell viability analyzed using a viability dye as the fraction of viable cells of the total cell population lifted from the well. (N = 5 mice) Statistical analysis performed using Welch’s T test for WT/KO and Paired T test for Iso/aPD-L1. (B) Log2 fold change of KO/WT and aPD-L1/iso of CD206 and Arg1 expression in M1 polarized BMDMs. (KO N = 5 mice, Blockade N = 3 mice) Statistical analysis performed using Welch’s T test. (C) Log2 fold change of KO/WT and aPD-L1/iso of iNOS, CD206, and Arg1 expression in TCM polarized BMDMs. (KO N = 7 mice Blockade N = 6 mice) Statistical analysis performed using Welch’s T test.

Supplementary Figure 3 (A) Flow cytometry analysis performed to confirm blockade of PD-L1 in the 96hr and post-polarization M1 polarized treatment groups. (N = 4 mice) Statistical analysis was performed using a Lognormal ordinary one-way ANOVA with Tukey’s multiple comparisons Test. (B) Baseline expression of M1 polarization markers in 96hr and post-polarization groups. (M0 N = 3 mice, M1 N = 4 mice, TCM 96hr Polarization N = 4 mice, TCM Post-Polarization N = 7 mice). Statistical analysis was performed using Mixed-effects analysis with Tukey’s multiple comparisons. (C) Baseline expression of TCM polarization markers in 96hr and post-polarization groups. (M0 N = 3 mice, M1 N = 4 mice, TCM 96hr Polarization N = 4 mice, TCM Post-Polarization N = 7 mice). Statistical analysis was performed using Mixed-effects analysis with Tukey’s multiple comparisons. (D) Polarization marker expression of naïve BMDMs in the 96hr treatment group (N = 3 mice). (E) Polarization marker expression of naïve BMDMs in the post-polarization treatment group (N = 3 mice). The remaining statistical analysis was performed with Ratio paired T test.

Supplementary Figure 4 (A) Flow cytometry analysis of effects of blockade on surface and total (surface and intracellular stain) levels of CD80 expression in M1 and TCM BMDMs treated with Isotype or PD-L1 blockade. (N = 3 mice). Statistics for analysis done with full effect 2-way ANOVA with Sidaks corrected multiple comparisons *post-hoc* test on lognormal data. (B) Analysis of CD80 transcript expression in M1 and TCM polarized BMDMs upon PD-L1 blockade and knockout. (N = 3 mice). Statistics for analysis done with two-tailed Welch’s corrected t test for WT vs KO and paired t test for Iso vs Blockade. (C) Flow cytometry analysis of effects of blockade on surface and total (surface and intracellular stain) levels of PD-L1 expression in M1 and TCM BMDMs treated with Isotype or PD-L1 blockade. (N = 3 mice). Statistics for analysis done with full effect 2-way ANOVA with Sidaks corrected multiple comparisons *post-hoc* test on lognormal data. (D) Analysis of PD-L1 transcript expression in M1 and TCM polarized BMDMs upon PD-L1 blockade (N = 3 mice). Statistics for analysis performed with two tailed welch’s paired t test.

Supplementary Figure 5 (A) Baseline expression of antigen presentation and checkpoint markers of WT BMDMs. (PD-1 N = 3, M0 N = 7, M1 N = 5, TCM N = 7) Statistical analysis was performed using Mixed-effects analysis with Tukey’s multiple comparisons. (B) Baseline expression of antigen presentation and checkpoint markers of Isotype treated BMDMs. (PD-1 N = 3 M0 N = 6, M1 N = 3, TCM N = 6) Statistical analysis was performed using Mixed-effects analysis with Tukey’s multiple comparisons. (C) Antigen presentation and checkpoint marker expression of WT/KO naïve BMDMs. (PD-1 N = 3 WT/KO N = 7). (D) Antigen presentation and checkpoint marker expression of Iso/aPD-L1 naïve BMDMs. (PD = 1 N = 3 Iso/aPD-L1 N = 6). C and D statistical analysis performed with Welch’s T test analysis of Log10 transformed data for WT/KO analysis and Ratio paired T test for Iso/aPD-L1 analysis.

Supplementary Figure 6 (A) Comparison of KO and anti-PD-L1 surface level fold changes of MHCI in M1 and TCM polarization states. (M1 KO N = 5 mice, M1 aPD-L1 N = 6 mice, TCM KO N = 7 mice, TCM aPD-L1 N = 9 mice) Statistical analysis was performed using two-tailed T tests. (B) Comparison of KO and anti-PD-L1 surface level fold changes of PD-L1 in M1 and TCM polarization states. (M1 KO N = 5 mice, M1 aPD-L1 N = 6 mice, TCM KO N = 7 mice, TCM aPD-L1 N = 9 mice). Statistical analysis was performed using two-tailed T tests. (C) Analysis of Pdcd1 transcript expression in M1 polarized BMDMs upon PD-L1 blockade and comparison of effects of blockade on surface and total levels of PD-1 (N = 3 mice). (D) Representative flow plots of PD-1 Full Minus One controls with M1 and TCM polarized BMDMs with Full stains. (E) Comparison of KO and anti-PD-L1 surface level fold changes of PD-1 in M1 and TCM polarization states. (KO N = 3 mice, aPD-L1 N = 6 mice). Statistical analysis was performed using two-tailed T tests. (F) Analysis of PD-L2 transcript expression in TCM polarized BMDMs upon PD-L1 blockade and comparison of effects of blockade on surface and total levels of PD-L2 (N = 3 mice).
