# Supplementary material for: PD-L1 deletion or blockade intrinsically reduces macrophage CD80 levels
Source: Front Oncol. 2026 Oct 30;16:1917545. doi: 10.3389/fonc.2026.1917545 (PMC13759293; doi:10.3389/fonc.2026.1917545)
Supplement: Supplementary file 1 [file DataSheet1.pdf]

PD-L1 deletion or blockade intrinsically reduces macrophage CD80 levels

Trinity Waddell, Haidong Dong, Minna Roh-Johnson, Jessica N. Lancaster

Supplementary Data

Supplementary Figure 1

Supplementary Figure 2

Supplementary Figure 3

Supplementary Figure 4

Supplementary Figure 5

Supplementary Figure 6

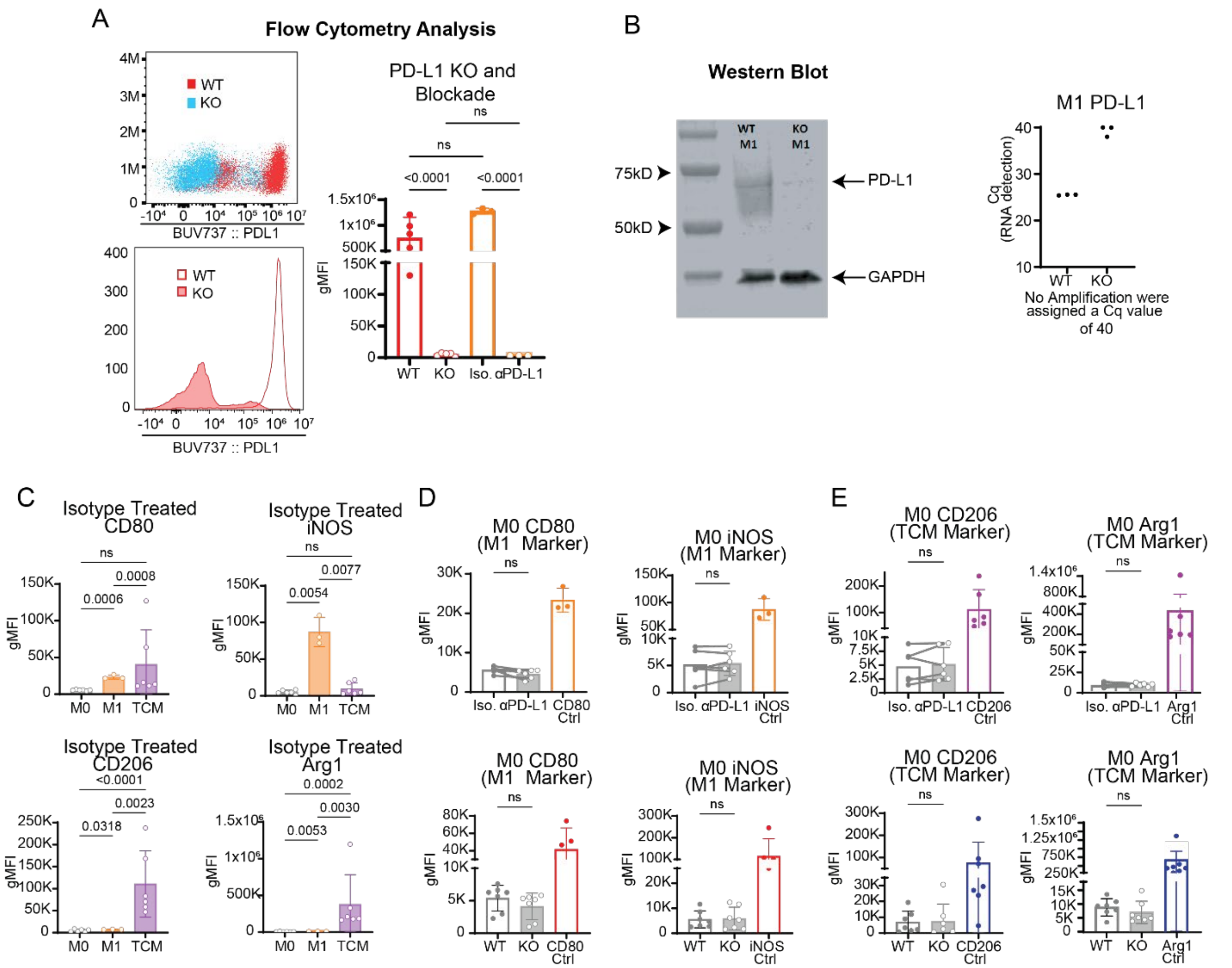

Supplementary Figure 1

(A) PD-L1 knockout and blockade of M1 BMDMs as confirmation of knockout and complete blockade through flow cytometry. (KO N=5 mice, Blockade N=3 mice). Statistical analysis was performed using a Lognormal ordinary one-way ANOVA with Tukey's multiple comparisons Test.

(B) Western blot, and qPCR (right figure) of M1 BMDMs as confirmation of PD-L1 knockout (Western Blot N=1 mouse, qPCR N=3 mice). For RNA analysis non-amplified replicates were assigned a raw Cq of 40. Each dot represents an averaged technical duplicate of a single mouse.

(C) Baseline expression of polarization panel in Isotype treated BMDMs. CD80 TCM expression appears higher than M1 BMDMs due to experimental day variability resulting in outliers. Experimental day matched samples result in M1 BMDMs with the highest expression (M0 N=6 mice, M1 N=3 mice, TCM N=6 mice). Statistical analysis was performed using Mixed-effects analysis with Tukey's multiple comparisons.

(D) M1 marker expression of naïve BMDMs WT/KO and Iso/aPD-L1. (Blockade N=6 mice, KO N=7 mice). Statistical analysis was performed with Welch's T test analysis of

Log10 transformed data for WT/KO analysis and Ratio paired T test for Iso/aPD-L1 analysis.

(E) TCM marker expression of naïve BMDMs WT/KO and Iso/aPD-L1 (Blockade N=6 mice, KO N=7 mice). The remaining statistical analysis was performed with Welch's T test analysis of Log10 transformed data for WT/KO analysis and Ratio paired T test for Iso/aPD-L1 analysis.

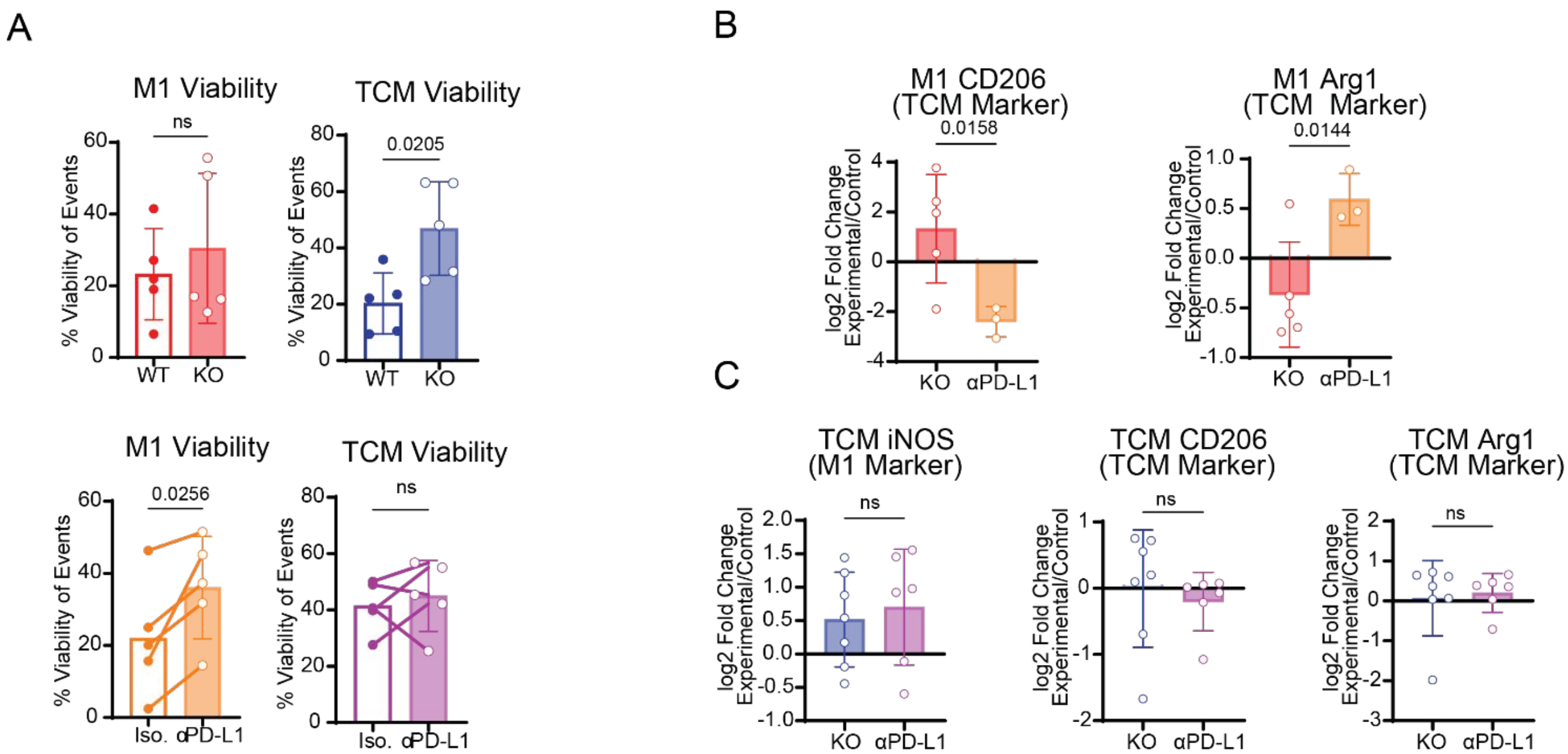

Supplementary Figure 2

(A) Cell viability analyzed using a viability dye as the fraction of viable cells of the total cell population lifted from the well. (N=5 mice) Statistical analysis performed using Welch's T test for WT/KO and Paired T test for Iso/aPD-L1.

(B) Log2 fold change of KO/WT and aPD-L1/iso of CD206 and Arg1 expression in M1 polarized BMDMs. (KO N=5 mice, Blockade N=3 mice) Statistical analysis performed using Welch's T test.

(C) Log2 fold change of KO/WT and aPD-L1/iso of iNOS, CD206, and Arg1 expression in TCM polarized BMDMs. (KO N=7 mice Blockade N=6 mice) Statistical analysis performed using Welch's T test.

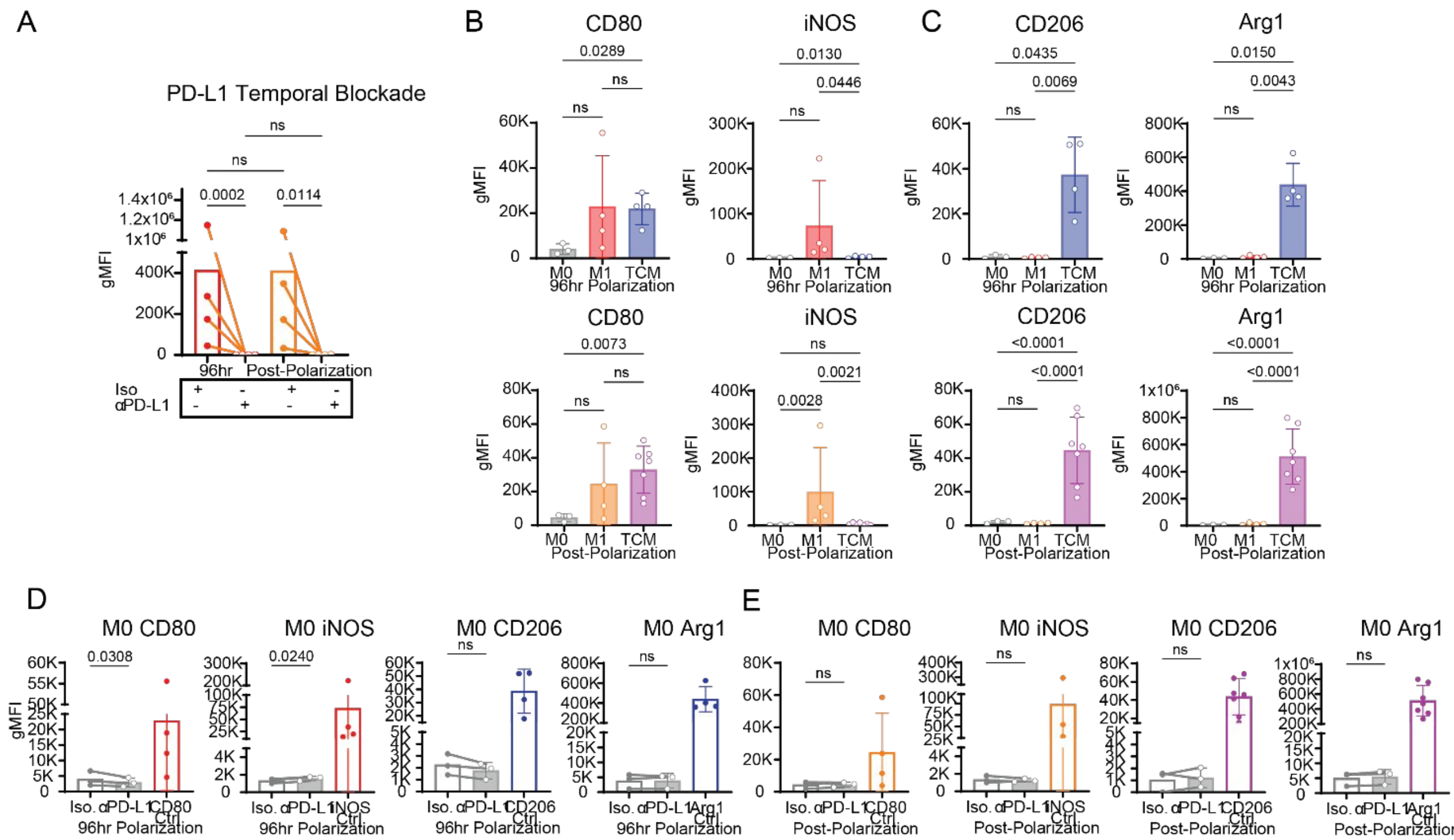

Supplementary Figure 3

(A) Flow cytometry analysis performed to confirm blockade of PD-L1 in the 96hr and post-polarization M1 polarized treatment groups. (N=4 mice) Statistical analysis was performed using a Lognormal ordinary one-way ANOVA with Tukey's multiple comparisons Test.

(B) Baseline expression of M1 polarization markers in 96hr and post-polarization groups. (M0 N=3 mice, M1 N=4 mice, TCM 96hr Polarization N=4 mice, TCM Post-Polarization N=7 mice). Statistical analysis was performed using Mixed-effects analysis with Tukey's multiple comparisons.

(C) Baseline expression of TCM polarization markers in 96hr and post-polarization groups. (M0 N=3 mice, M1 N=4 mice, TCM 96hr Polarization N=4 mice, TCM Post-Polarization N=7 mice). Statistical analysis was performed using Mixed-effects analysis with Tukey's multiple comparisons.

(D) Polarization marker expression of naïve BMDMs in the 96hr treatment group (N=3 mice).

(E) Polarization marker expression of naïve BMDMs in the post-polarization treatment group (N=3 mice). The remaining statistical analysis was performed with Ratio paired T test.

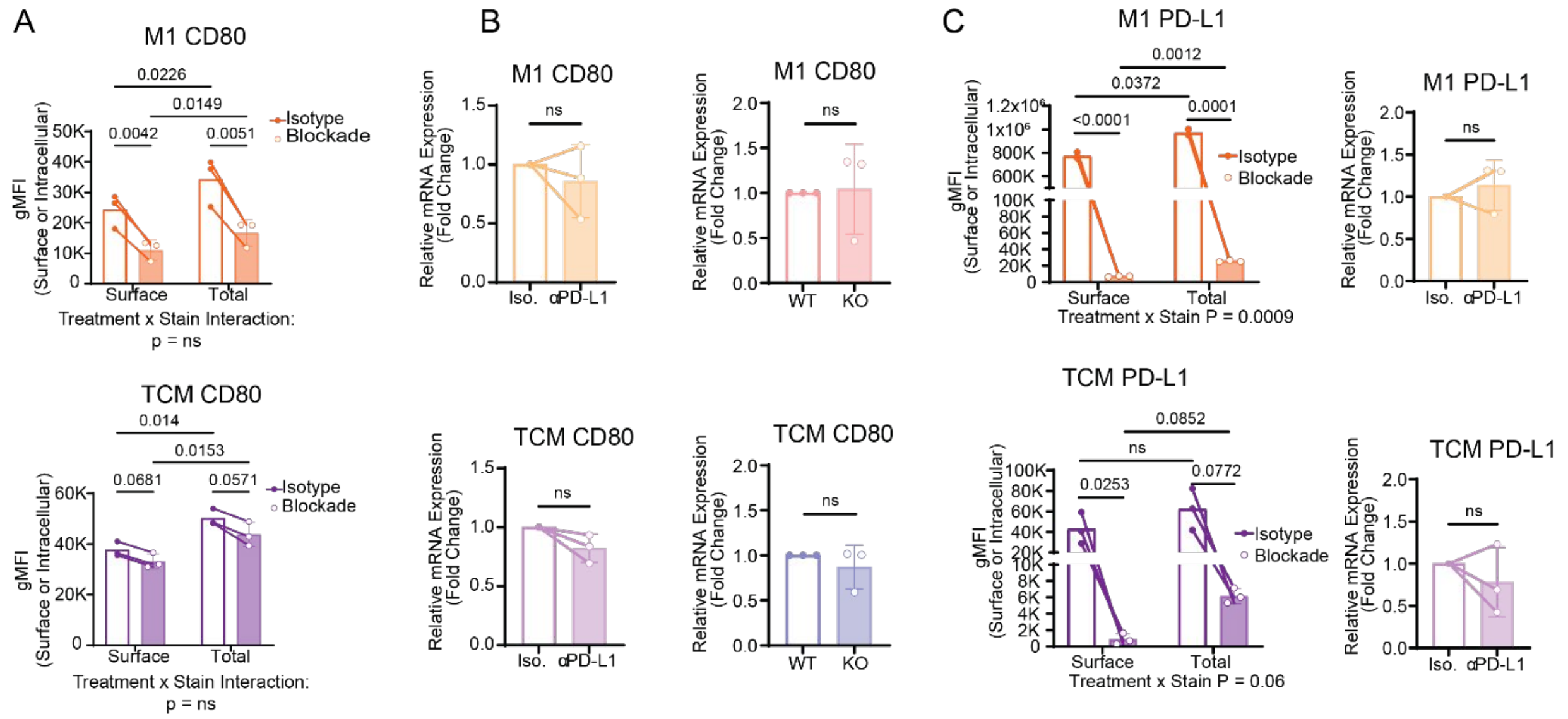

Supplementary Figure 4

(A) Flow cytometry analysis of effects of blockade on surface and total (surface and intracellular stain) levels of CD80 expression in M1 and TCM BMDMs treated with Isotype or PD-L1 blockade. (N=3 mice). Statistics for analysis done with full effect 2-way ANOVA with Sidaks corrected multiple comparisons post-hoc test on lognormal data.

(B) Analysis of CD80 transcript expression in M1 and TCM polarized BMDMs upon PD-L1 blockade and knockout. (N=3 mice). Statistics for analysis done with two-tailed Welch's corrected t test for WT vs KO and paired t test for Iso vs Blockade.

(C) Flow cytometry analysis of effects of blockade on surface and total (surface and intracellular stain) levels of PD-L1 expression in M1 and TCM BMDMs treated with Isotype or PD-L1 blockade. (N=3 mice). Statistics for analysis done with full effect 2-way ANOVA with Sidaks corrected multiple comparisons post-hoc test on lognormal data.

(D) Analysis of PD-L1 transcript expression in M1 and TCM polarized BMDMs upon PD-L1 blockade (N=3 mice). Statistics for analysis performed with two tailed welch's paired t test.

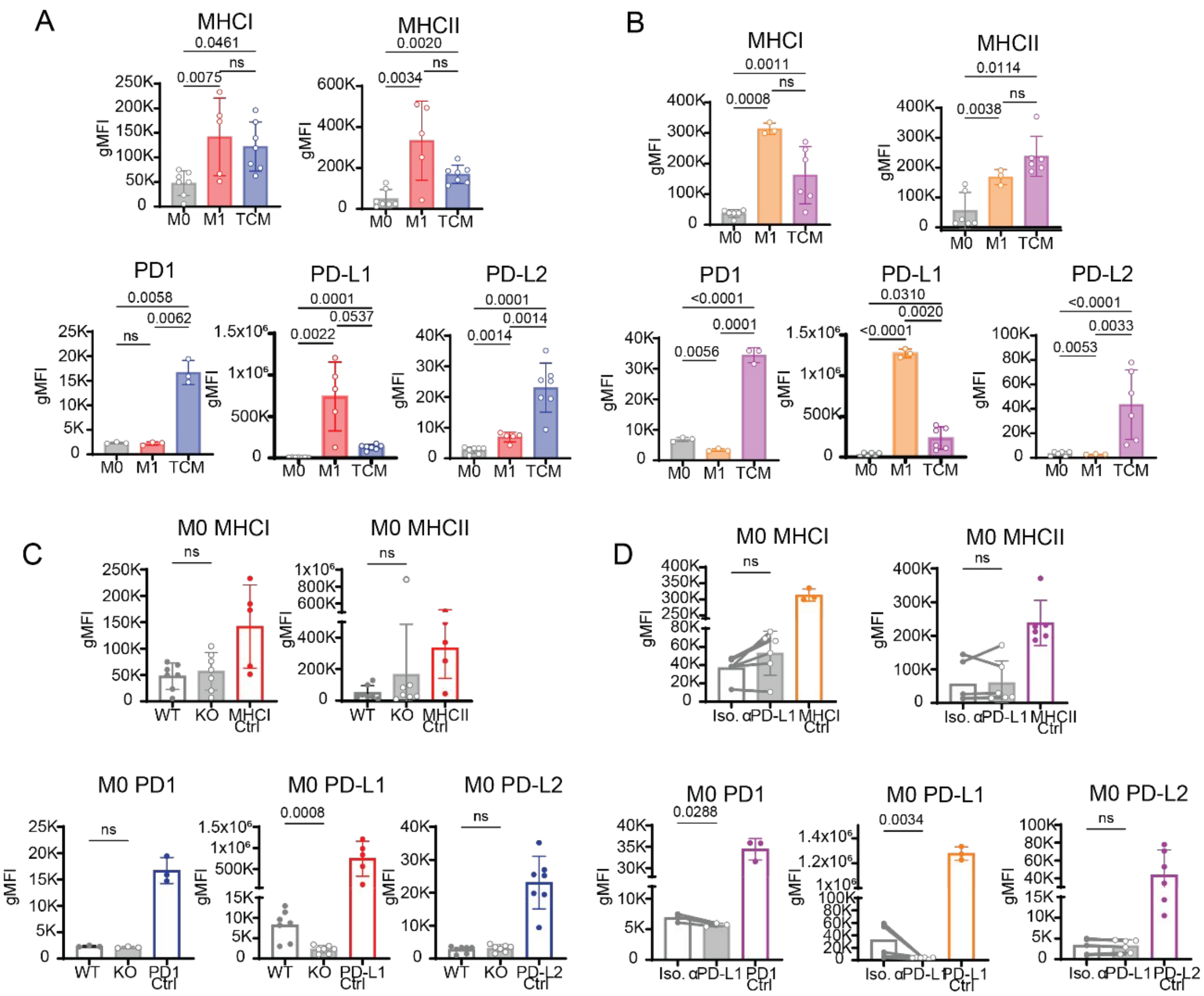

Supplementary Figure 5

(A) Baseline expression of antigen presentation and checkpoint markers of WT BMDMs. (PD-1 N=3, M0 N=7, M1 N=5, TCM N=7) Statistical analysis was performed using Mixed-effects analysis with Tukey's multiple comparisons.

(B) Baseline expression of antigen presentation and checkpoint markers of Isotype treated BMDMs. (PD-1 N=3 M0 N=6, M1 N=3, TCM N=6) Statistical analysis was performed using Mixed-effects analysis with Tukey's multiple comparisons.

(C) Antigen presentation and checkpoint marker expression of WT/KO naïve BMDMs. (PD-1 N=3 WT/KO N=7).

(D) Antigen presentation and checkpoint marker expression of Iso/aPD-L1 naïve BMDMs. (PD=1 N=3 Iso/aPD-L1 N=6). C and D statistical analysis performed with Welch's T test analysis of Log10 transformed data for WT/KO analysis and Ratio paired T test for Iso/aPD-L1 analysis.

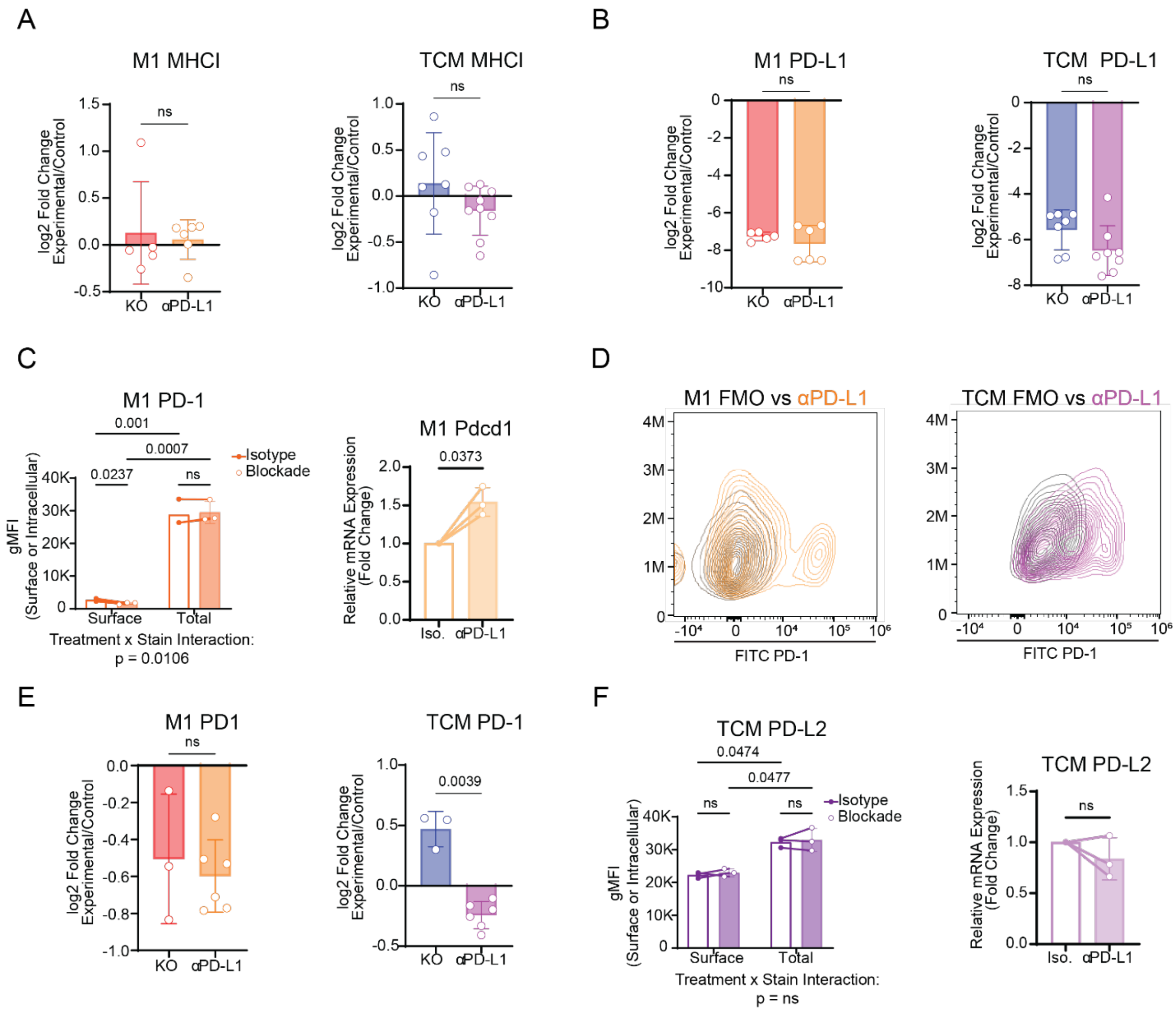

Supplementary Figure 6

(A) Comparison of KO and anti-PD-L1 surface level fold changes of MHCI in M1 and TCM polarization states. (M1 KO N=5 mice, M1 aPD-L1 N=6 mice, TCM KO N=7 mice, TCM aPD-L1 N=9 mice) Statistical analysis was performed using two-tailed T tests.

(B) Comparison of KO and anti-PD-L1 surface level fold changes of PD-L1 in M1 and TCM polarization states. (M1 KO N=5 mice, M1 aPD-L1 N=6 mice, TCM KO N=7 mice, TCM aPD-L1 N=9 mice). Statistical analysis was performed using two-tailed T tests.

(C) Analysis of Pdcd1 transcript expression in M1 polarized BMDMs upon PD-L1 blockade and comparison of effects of blockade on surface and total levels of PD-1 (N=3 mice).

(D) Representative flow plots of PD-1 Full Minus One controls with M1 and TCM polarized BMDMs with Full stains.

(E) Comparison of KO and anti-PD-L1 surface level fold changes of PD-1 in M1 and TCM polarization states. (KO N=3 mice, aPD-L1 N=6 mice). Statistical analysis was performed using two-tailed T tests.

(F) Analysis of PD-L2 transcript expression in TCM polarized BMDMs upon PD-L1 blockade and comparison of effects of blockade on surface and total levels of PD-L2 (N=3 mice).
